# Anatomical Study and Comprehensive Review of the Incisivus Labii Superioris Muscle: Application to Lip and Cosmetic Surgery

**DOI:** 10.7759/cureus.1689

**Published:** 2017-09-15

**Authors:** Joe Iwanaga, Koichi Watanabe, Cameron K Schmidt, Vlad Voin, Fernando Alonso, Rod J Oskouian, R. Shane Tubbs

**Affiliations:** 1 Seattle Science Foundation; 2 Department of Anatomy, Kurume University School of Medicine; 3 Clinical Anatomy, Seattle Science Foundation; 4 Research Fellow, Seattle Science Foundation; 5 Neurosurgery, University Hospitals of Cleveland, Case Medical Center; 6 Neurosurgery, Complex Spine, Swedish Neuroscience Institute; 7 Neurosurgery, Seattle Science Foundation

**Keywords:** anatomy, cleft lip, facial muscles, lip, cosmetic rhinoplasty, botox

## Abstract

Objectives

The incisivus labii superioris muscle, which originates from the floor of the incisive fossa of the maxilla, has previously been described, it is not well understood. The purpose of this study was to investigate the incisivus labii superioris muscle with detailed dissection.

Methods

Twenty-six halves from thirteen fresh frozen cadaveric Caucasian heads were used in this study. First, the incisivus labii superioris muscle was dissected to reveal its origin and insertion, and its relationship to other mimetic muscles. Secondly, the distance from the midline to the innermost part of the bony attachment of the muscle was measured. The literature describing the incisivus labii superioris muscle was reviewed.

Results

The incisivus labii superioris muscle consisted of two parts, inferior and superior. The former merged into the orbicularis oris and the latter into the nasalis. The mean distance from the midline to the innermost part of the bony attachment of the incisivus labii superioris muscle was 4.8 ± 1.7 mm on the right side and 4.9 ±1.7 mm on the left.

Conclusions

The results of the present study suggest that the inferior part of the incisivus labii superioris should be considered as an accessory muscle of the orbicularis oris complex, and the superior part is the nasalis muscle.

## Introduction

**Figure 1:**
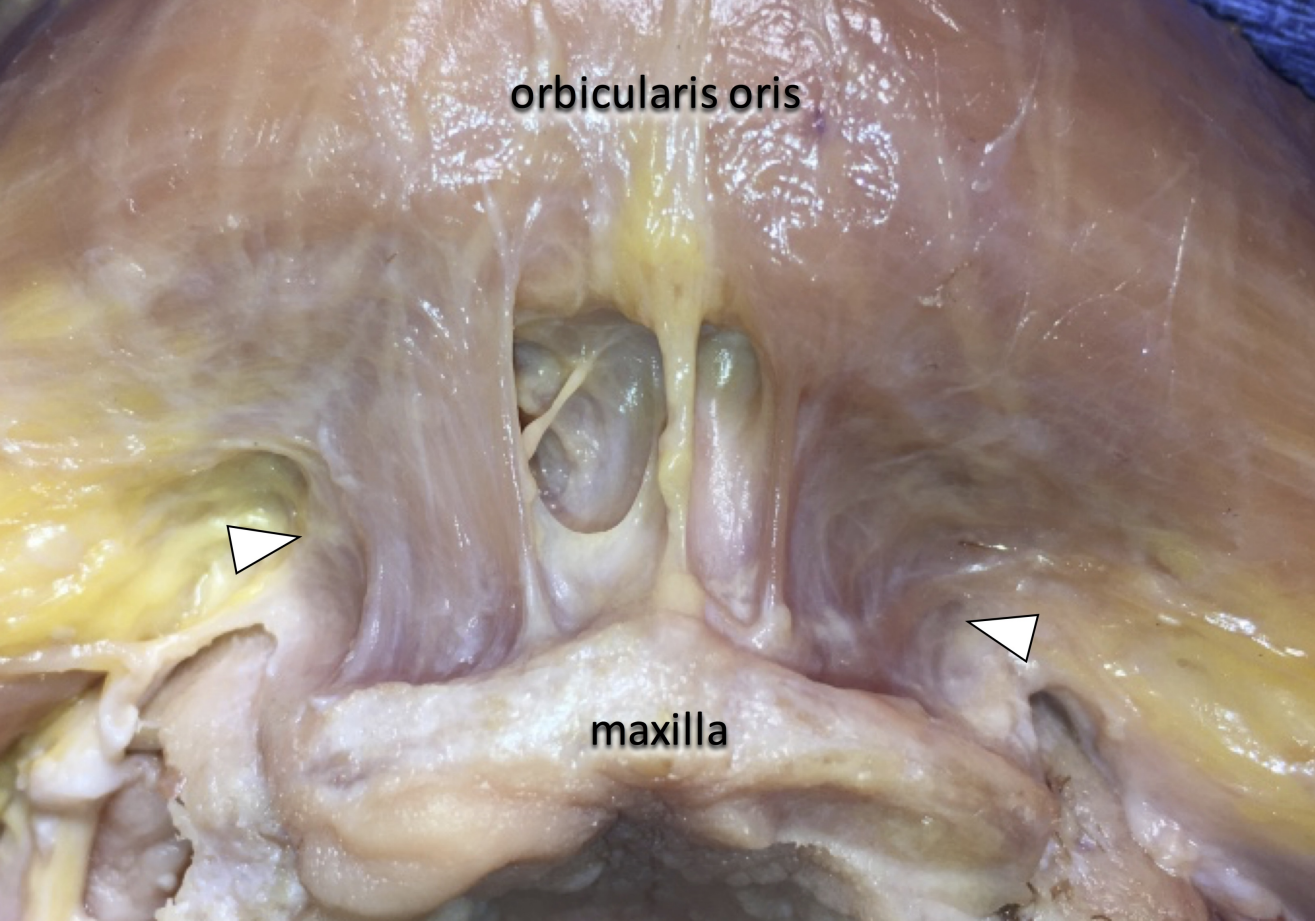
Bilateral muscle fibers (arrowheads), which originate from the maxillary bone vertically towards the orbicularis oris.

The upper lip, except for the corner of the mouth, mainly consists of three kinds of mimetic muscle: the orbicularis oris (OO) in the upper lip, the levator labii superioris (LLS), and the levator labii superioris alaeque nasi (LLSAN). During our dissection, we found bilateral muscle fibers originating from the maxillary bone and traveling vertically to the OO (Figure 1).

**Figure 2:**
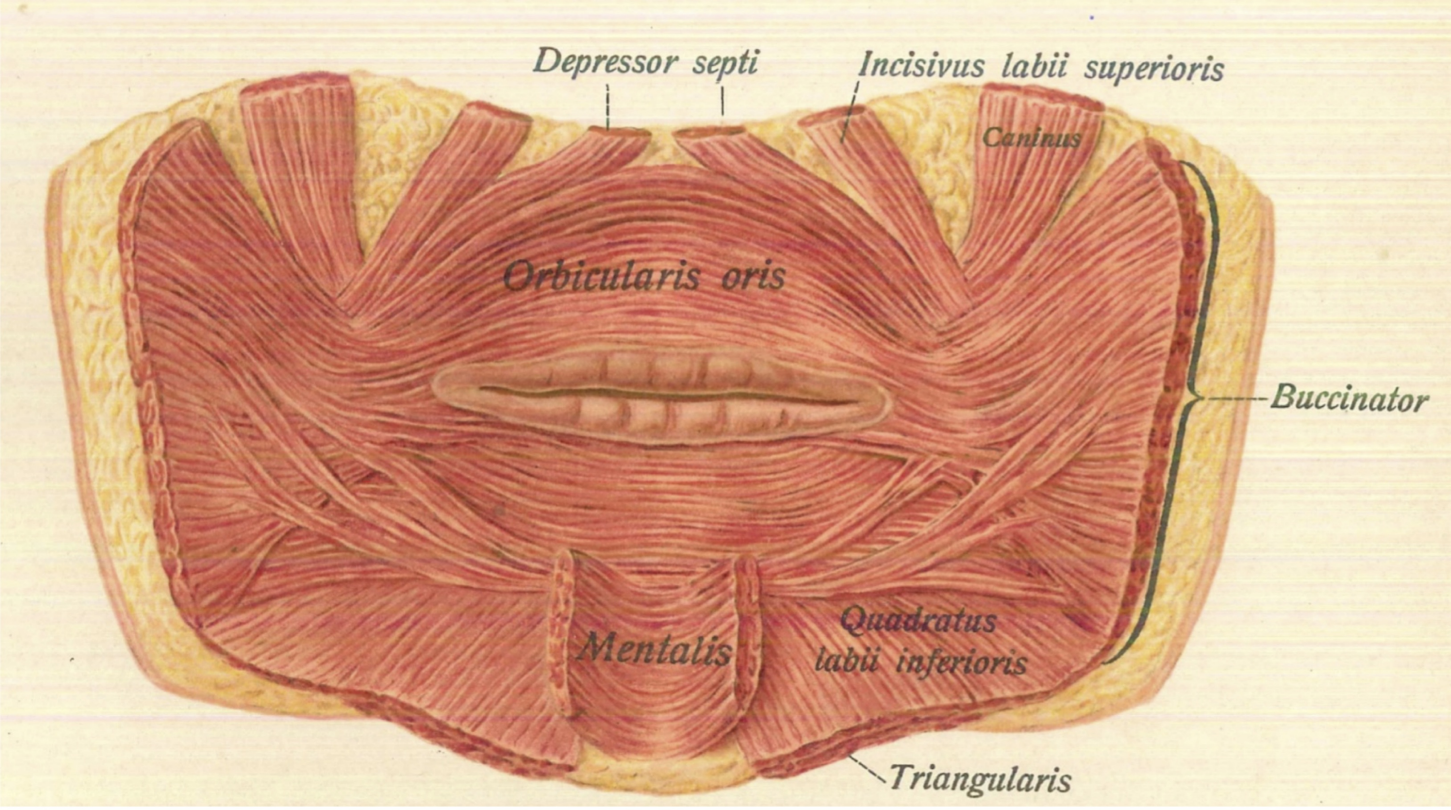
Posterior view of the incisivus labii superioris muscle. [4]

An incisivus labii superioris muscle (ILS) has been described, originating from the floor of the incisive fossa of the maxilla and running lateral and parallel to the OO [1]. Others have mentioned that the OO in the upper lip has a bony attachment above the upper incisor [2], or a bony attachment in the anterior midline of the maxilla [3]. Also, some literature depicted this muscle [4] (Figure 2).

Thus, there seems to be no consensus about the detailed muscular anatomy and terminology of the upper labial region, especially the bony attachment of the muscles that some researchers regard as the ILS. To make matters worse, most illustrations have shown the outline of the mimetic muscles as seen from the anterior or lateral perspectives and many of them include only drawings of the bony attachment of the ILS [1], though Burkitt portrayed the ILS as seen from behind [5]. Thus, even though several textbooks include overviews of the ILS, to our knowledge, its detailed anatomy and its relationship to surrounding structures have never been investigated. The purpose of this study was to investigate the ILS muscle, elucidate its relationship to adjacent mimetic muscles, and establish the correct terminology by anatomical study and literature review.

## Materials and methods

Anatomical study

Twenty-six halves from thirteen fresh, frozen cadaveric Caucasian heads were used in this study. The specimens were derived from seven males and six females and their age at death ranged from 58 to 99 years (mean age; 79.8 ± 11.7 years). The initial incision was made into the mucogingival junction horizontally from the right first premolar to the left first premolar, and then a transverse incision was made into the mucodermal junction of the upper lip from the right to the left corner of the mouth.

Next, the mucosa that covered the OO was elevated from the site of the transverse incision towards the initial horizontal incision at the mucogingival junction. The relationship between the folds of the mucosa and the ILS was observed, the outline of the ILS was recorded to reveal its origin and insertion; its relationship to the OO and the other mimetic muscles was also observed. Finally, the ILS was cut at its origin (maxillary bone), the location was recorded, and the circumference of the bony attachment and the distance from the midline to the innermost part of the bony attachment of the muscle was measured using micro calipers (Mitutoyo, Kanagawa, Japan).

Comprehensive literature review

To correct the terminology concerning the ILS, the literature and textbooks that included descriptions of the bony attachment of the “orbicularis oris”, “nasalis” and “incisivus labii superioris muscle” were reviewed. The origin, insertion, terminology, characteristics, relationship to adjacent mimetic muscles and the action of a muscle that had a bony attachment to an incisor or canine tooth of the maxilla were assessed to establish an appropriate new terminology for the ILS.

The protocol of the present study did not require approval by an ethics committee at our institutions, and the work was performed in accordance with the requirements of the Declaration of Helsinki (64th World Medical Association (WMA) General Assembly, Fortaleza, Brazil, October 2013). 

## Results

Anatomical study

**Figure 3:**
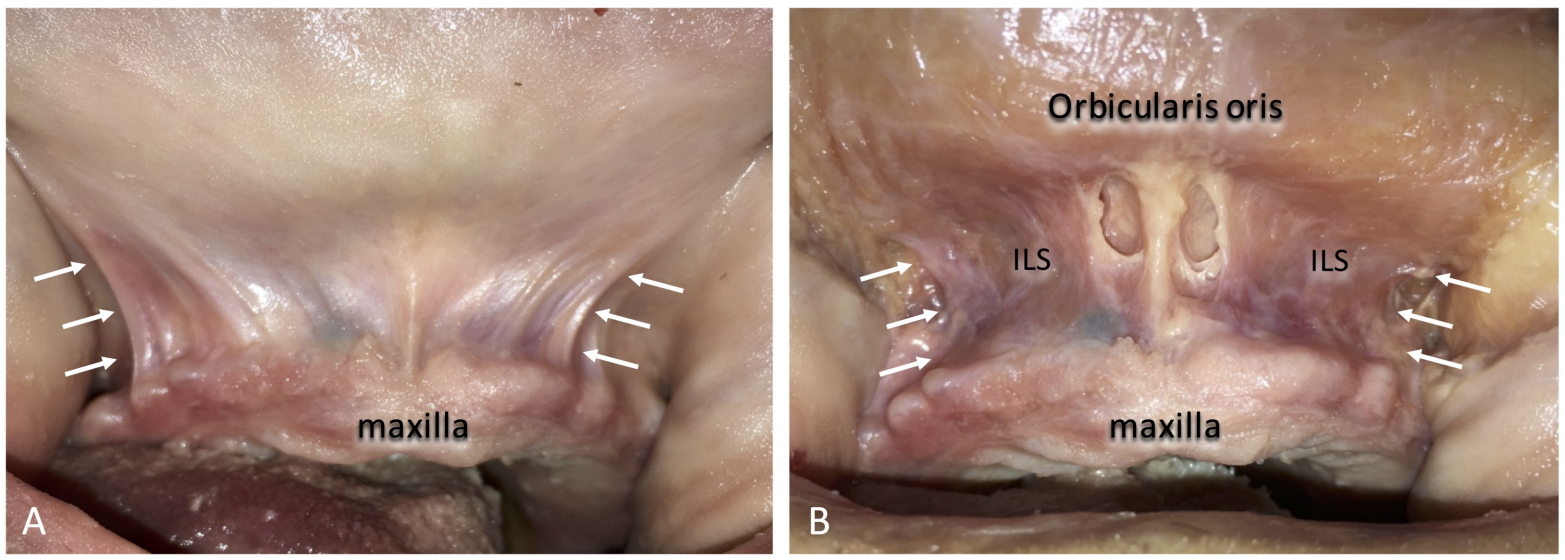
The lateral border of the inferior part of the incisivus labii superioris muscle (ILS) corresponds to the upper buccal frenulum (arrows). ILS; incisivus labii superioris muscle. A: Before incision, B: ILS following the removal of the mucosa.

The ILS consisted of two parts, inferior and superior. The lateral border of the inferior part of the ILS (i-ILS) corresponded to the upper buccal frenulum and merged into the OO. Despite its thickness and width differed among specimens, the i-ILS mainly went straight to and joined the OO, while small parts went laterally parallel to and joined the OO. It formed the roof of the anterior part of the oral vestibule. Underneath the mucosa of the upper labial frenulum, there was tight connective tissue on the midline of the maxilla from the OO, which was right behind the philtrum, to the periosteum just above the mucogingival junction (Figure 3).

**Figure 4:**
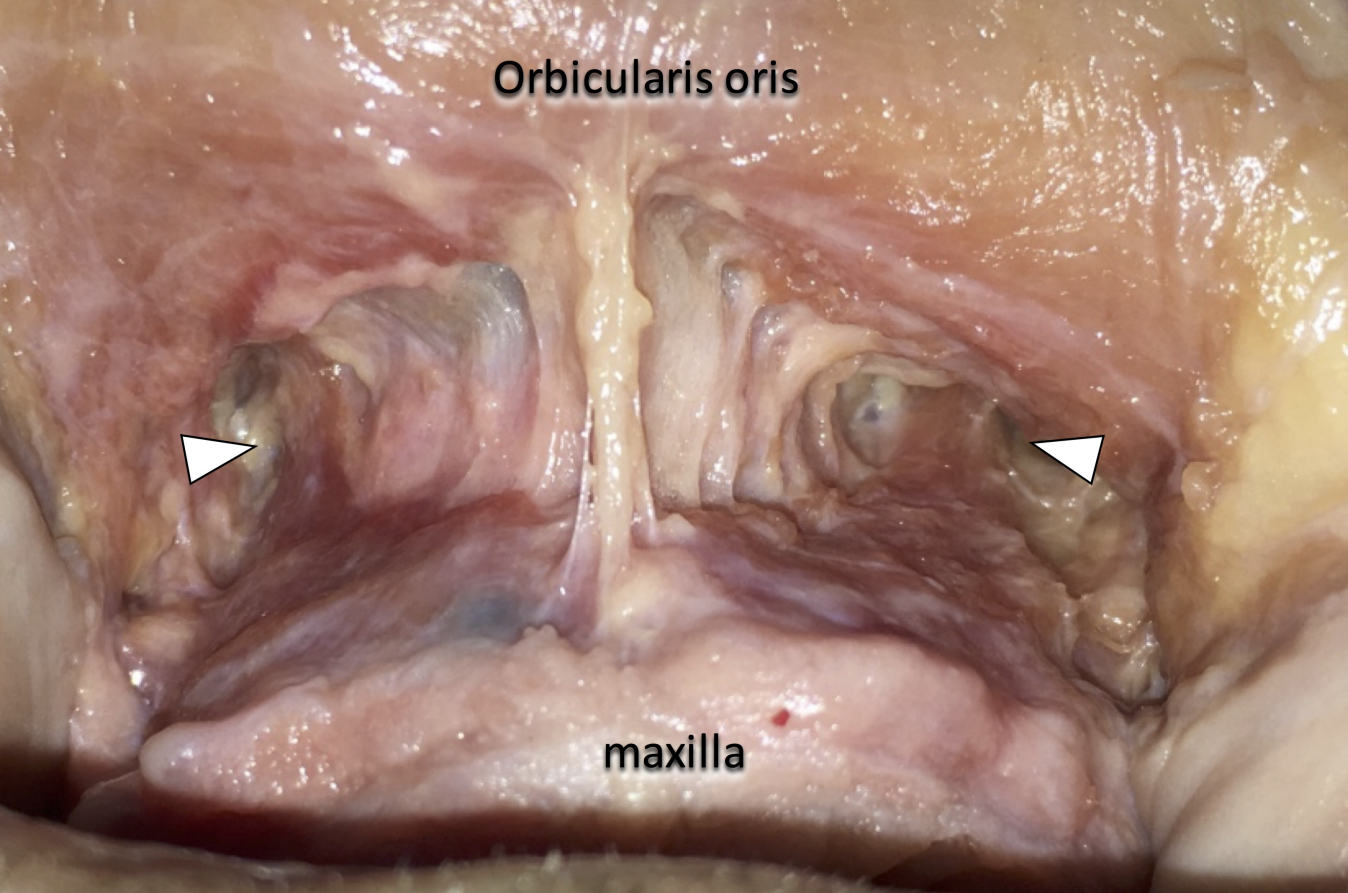
The superior part of the incisivus labii superioris muscle (arrowheads) went upwards underneath the inferior part of the incisivus labii superioris muscle and the orbicularis oris.

After the ILS was exposed, its superior part (s-ILS) went upwards underneath the i-ILS and the OO (Figure 4).

**Figure 5:**
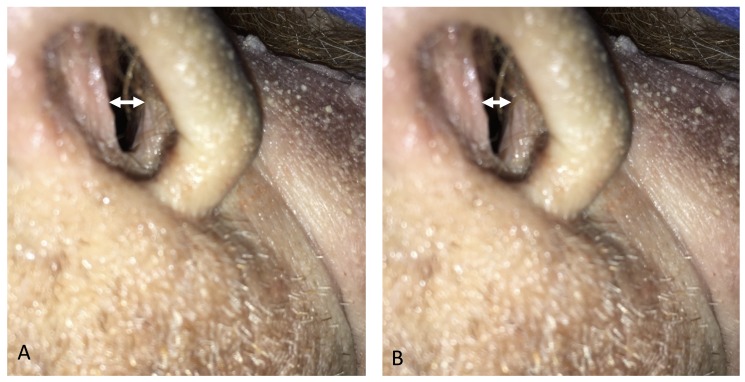
The superior part of the incisivus labii superioris muscle (nasalis) makes the naris vestibule open (A) and close (B). A: retracting the muscle, B: releasing the muscle.

Although a few fibers of the s-ILS merged into the LLS and LLSAN, most of the muscle merged into the alar and transverse part of the nasalis, and its function was to shrink the naris vestibule (Figure 5).

**Figure 6:**
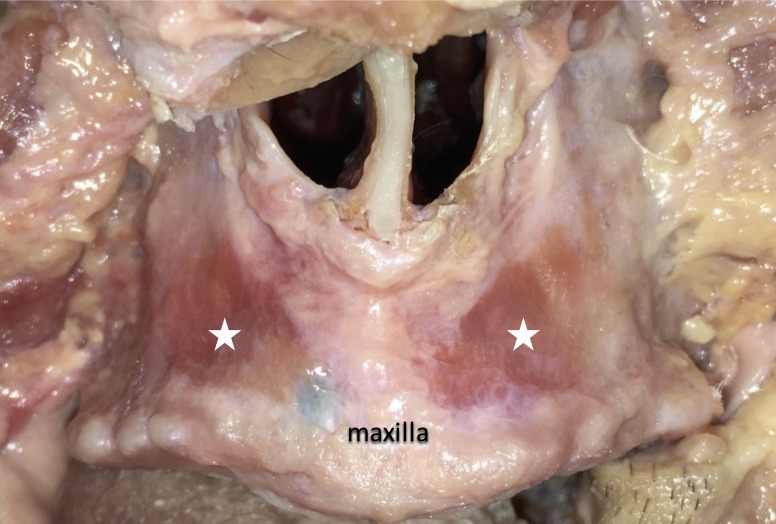
The bony attachment of the incisivus labii superioris muscle ( represented by white stars).

The location of the bony attachment of the ILS ranged from the central incisor medially to the canine laterally; the outline was triangular, and the inferior border of the bony attachment corresponded to the mucogingival junction in all the subjects (Figure 6).

In all twenty-six halves, the i-ILS and s-ILS had a common origin. In one specimen, the i-ILS attachments split in two, bilaterally. The superficial bony attachments were not similarly split but were continuous throughout. The ILS muscles joined together deep to the superficial separation.

**Figure 7:**
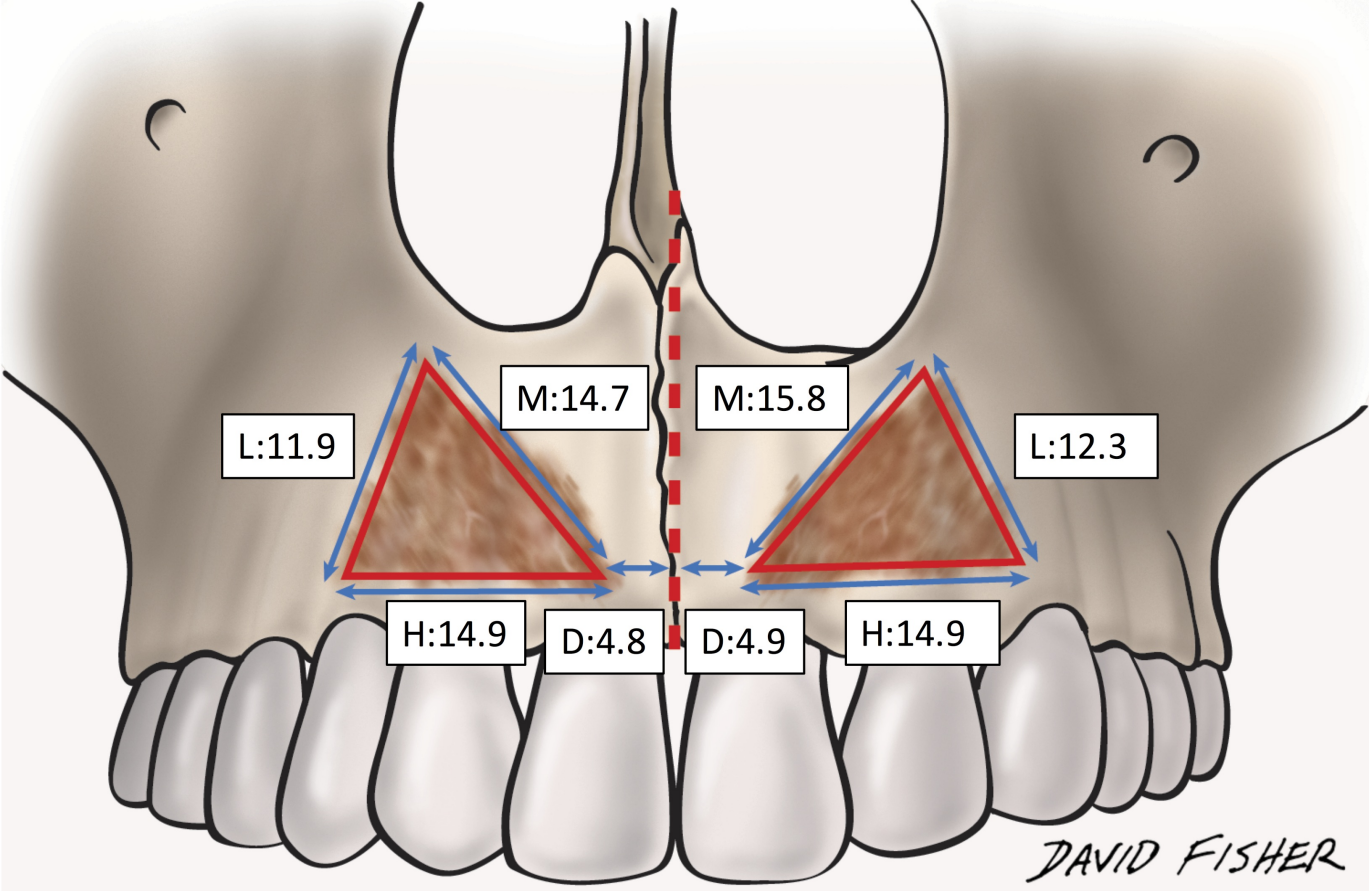
The measurement of the distance and circumference of the bony attachment. D: the distance from mid-line to innermost part of the bony attachment, H: length of horizontal part, L: length of lateral part, M: length of medial part (mm).

The mean lengths of the horizontal, medial and lateral parts of the bony attachment on the right side were 14.9 ± 2.9 mm (range 11.7 to 20.5 mm), 14.7 ± 3.0 mm (range 6.9 to 18.2 mm), and 11.9 ± 3.4 mm (from 5.7 to 16.0 mm), respectively, and on the left side were 14.9 ± 3.2 mm (range 11.2 to 21.4 mm), 15.8 ± 3.7 mm (range 11.6 to 23.3 mm) and 12.3 ± 3.7 mm (range 7.8 to 18.9 mm), respectively. The mean distance from the midline to the innermost part of the bony attachment of the ILS was 4.8 ± 1.7 mm (range 1.8 to 7.9 mm) on the right side and 4.9 ± 1.7 mm (range 2.5 to 8.3 mm) on the left (Figure 7).

After the muscle and periosteum were removed, no fossa or tubercle was identified except the canine fossa on the maxilla.

Comprehensive literature and textbook review

Anatomical manuscripts and texts published between 1887 [6] and 2015 [1] were reviewed.

Bony attachment

The ILS originates from the alveolar process [7], the alveolar border of the maxilla [8], the floor of the incisive fossa of the maxilla above the eminence of the upper lateral incisor [1, 9], the alveolar process of the upper incisor [6], the alveolar juga of the upper canine [10-12], the alveolar juga of the upper lateral incisors [4, 13], the maxillary bone above the incisor [14], the incisive fossae of the maxillary bone [15-17], or the maxilla near the upper lateral incisor [18].

Course and insertion

This muscle courses laterally, closely following the peripheral bundles of the OO [7], passes from the sides of the lips [10], arching laterally and is continuous with the other muscles at the angle of the mouth [8] including the deeper part of the orbicularis oris [13], besides blending with the muscles passing to the mouth [15]; it passes downwards and outwards to mingle with the other fibers of the orbicularis at the angles of the mouth [16] or the back of the orbicularis near the corner of the mouth [10], or passes laterally to insert into the corners of the mouth [11]. Initially, it lies deep to the superior part of orbicularis oris pars peripheralis. Arching laterally, its fiber bundles become intercalated between and parallel to the orbicular bundles. Approaching the modiolus, it segregates into superficial and deep parts; the former blends partially with the levator anguli oris and attaches to the body and apex of the modilous and the latter is attached to the superior cornu and the base of the modiolus [1], passes directly into the musculature of the orbicularis oris [4] and arch-like to the angle of the mouth and is interwoven there with the other muscles [12].

Terminology

Many different names have been used for the ILS, e.g., incisivus labii superioris [1, 4-5, 8, 10, 12-13, 16, 19], accessory fasciculi of the OO [14], facial portion of the OO [20], incisive bundles (muscles) of the OO [7, 18], and incisive Cowper [6]. It has been referred to as the accessory skeletal heads of the OO [7] and the accessory orbicular muscle [9]. It is described as slender slips [15], a small muscle bundle [10], flat, triangular and narrow in the form [12], and a small slender muscle [4]. It is powerfully developed in the Australian Aboriginal and well developed in the Sunda Islander [5].

Relationship to the adjacent mimetic muscles

The ILS is continuous with the other muscles at the angle of the mouth [8], the back of the orbicularis near the corner of the mouth [10], and above the angle of the mouth, and it is covered by the musculus M. quadratus labii superioris at the upper margin of the musculus M. orbicularis oris [12]. Each has a superficial and a deep insertion into the modiolus and is closely approximated to the OO [5]. Some of the lateral fibers of the ILS interlace with and have a common origin with those of the pars transversa M. nasalis, while others continue into it [5]. Still, others interlace with the pars alaris M. nasi, and the most medial fibers are continuous with those fibers of the M. orbicularis oris that extend upwards on to the septum [5]. The ILS has not been described as giving fibers to the nasal septum.

Action

The ILS draws the angle of the mouth medially and upwards [12].

## Discussion

Upper labial frenulum

The lip frenulum has been considered as bundle-like formations composed of fibrous, muscular or fibromuscular tissue and covered with a mucosa [20]. In the present study, all the specimens had tight connective tissue located from the midline of the maxilla to the OO, which formed the upper labial frenulum. Although no histological examination was conducted in the present study, we thought on the basis of the gross observation that the frenulum was formed only by this tight connective tissue and not by muscle. Some ILS fibers can be included in tissue specimens especially if the distance between the two sides of the medial part of the ILS is short. The function of the upper lip frenulum seemed to depend on the OO.

Definition of the incisivus labii superioris

The measurements in the present study showed that the bony attachment of the entire ILS was located in the alveolar process of the upper incisors (from the central incisor to the canine). This was as previously described. However, no publications have described the upper part of the bony attachment, which is the origin of the nasalis, and the triangular attachment. Our literature review revealed that some authors believed the ILS to be the bony heads or accessory bundle of the OO [7, 9, 14]. We agree, because all the fibers of the i-ILS join the OO, and regarding the lower lip, the incisivus labii inferioris is an accessory muscle of the OO complex [21-22]. No literature has mentioned the common bony attachment of the nasalis and i-ILS, but Burkitt and Lightoller [5] presented a similar illustration, a triangular bony attachment, but each attachment was separate in their study.

It is also interesting that the inferior border of the ILS corresponded to the mucogingival junction in all specimens. This means the ILS could define the border of the keratinized gingiva above the anterior maxillary teeth.

Only Spalteholz [12] mentioned the action of the ILS as it moves the angle of the mouth median wards and upwards. Our findings showed that most of the fibers of the i-ILS go anteriorly, so its main action is probably to retract the upper lip backward.

Surgical relevance

As this muscle is not generally known, we found no description of it in the surgical articles and textbooks we reviewed. Presumably, it is not considered during surgical procedures. Possible procedures affecting this muscle are as follows: upper labial vestibule incision to expose the maxillary bone, including maxillary bone fracture; correction of the gingival smile [23-24]; cleft lip repair [25]; and rhinoplasty [26].

During the labial vestibule incision, the vestibule is cut a little way up the labium side from the fornix to secure a margin to suture. It is cut toward the subperiosteal layer on the maxilla. In this procedure, the i-ILS is usually cut unknowingly, but some thin muscle slips that might be considered as this muscle can occasionally be observed. In regard to the gingival smile correction, Litton and Fournier [23] reported simply removing the labial vestibule mucosa and making the fornix shallow. Perhaps the muscle is removed along with the fornix mucosa. We considered that the result of this operation, a reduced lift of the upper lip, is obtained not only by making the fornix shallow but also by resection of this muscle. Miskinyar [24] proposed another method for the gingival smile. In outline, Miskinyar’s procedure is as follows. The incision is performed on the upper labial vestibule, the upper labium is lifted up and reflected, and the levator labii superior is amputated from behind. The force of elevating the upper lip will be weakened by this procedure and exposure of the gingiva can be prevented. During the procedure, the incisivus labii superioris is also cut at the labial vestibular incision; this enhances the result of the operation. Also in rhinoplasty [27], many surgeons transect the ILS to approach the depressor septi nasi muscle.

Cleft lip is an anomaly in which there is a cleft of some degree between the free margin of the upper lip and the nostril. In complete cleft cases, it is generally observed that the orbicularis oris muscle fibers run parallel to the cleft margins to the base of the ala on the diseased (lateral) side but attaches to the base of the columella on the healthy (medial) side [28-29]. From these descriptions [28], the attachment on the diseased side could be the incisivus labii superioris and those on the healthy side, the depressor septi. Also during cleft lip plasty, as represented by Millard’s procedure, the ala nasi is released from the muscle attachment and the cheek tissue on the diseased side is dissected widely on the epi-periosteal layer owing to the closure of the lip tissue defect [25]. We cannot be certain that those muscles are the incisivus labii superioris and depressor septi because it is currently difficult to perform anatomical dissection on cleft lip patients. However, considering the unclear description of the anatomy of the orbicularis oris in anatomical textbooks, there is room to reconsider the anatomy of the cleft lip.

Lastly, botulinum toxin is often used for aesthetic purposes on the face [30]. The anatomy of the orbicularis oris in this regard has been well discussed, but that of the ILS has not. To consider the complications and procedure, knowledge of this muscle is significant for surgeons prior to developing such injection procedures.

## Conclusions

This was the first comprehensive study of the “incisivus labii superioris” to include dissection photographs of this muscle. The results suggest that the inferior part of the “incisivus labii superioris” should be considered as an accessory muscle of the orbicularis oris (OO) complex, while the superior part is the nasalis. These two different parts of the muscle have a common origin of which the outline is triangular. Also, such a depiction of this incisivus labii superioris muscle (ILS) could potentially develop the new procedure of the cleft lip surgery and rhinoplasty in order to avoid unnecessary injury to this muscle.
